# Pericytes in the Development and Progression of Brain Diseases

**DOI:** 10.14336/AD.2025.0028

**Published:** 2026-04-20

**Authors:** Qingbin Wu, Xinyi Cui, Xiaochen Yuan, Jianqun Han, Hongwei Li, Ruijuan Xiu

**Affiliations:** ^1^Institute of Microcirculation, Chinese Academy of Medical Sciences & Peking Union Medical College, Beijing, 100005, China.; ^2^International Center of Microvascular Medicine, Chinese Academy of Medical Sciences, Beijing, 100005, China

**Keywords:** Microcirculation, Pericytes, Brain diseases, Blood-brain barrier

## Abstract

Pericytes are microvascular cells surrounding the endothelial cells on the outside of the capillaries in the body. They are crucial cells in the formation and ensure the integrity of vascular walls in the microcirculation. The pericytes enable that by regulating blood flow, maintaining the stability of the vascular wall, and ensuring the integrity of the blood-brain barrier (BBB). It has been confirmed that pericytes are involved in many brain diseases, including Alzheimer's disease, stroke, traumatic brain injury, epilepsy, brain tumors, brain tissue infection, and hypertension. These diseases are a huge burden on global health, and they exert significant strain on healthcare systems and society because of morbidity, mortality, and their impact on the overall quality of life of the affected population. This review study summarizes and evaluates the role of pericytes in the development and progression of many brain diseases and their role in disease progression regulation mechanisms, thereby providing new insights into the potential of pericytes in treating brain diseases.

## Introduction

Pericytes, which are also known as mural or rouget cells, refer to specialized cells that are located within the basement membrane of capillaries. They encapsulate the endothelial cells throughout the microcirculation of the body. The neurovascular unit (NVU) has these cells as its key components, crucial for brain health. The NVU consists of pericytes, smooth muscle cells, basement membrane, and vascular endothelial cells, forming the basic microvessel structure. Among the components, pericytes are mainly involved in microvascular constriction and are critical in blood flow regulation.

The anatomical and physiological functions of the NVU are dependent on pericytes. They participate in the determination of the stability of the vascular wall, integrity of the blood-brain barrier (BBB), and microcirculation. These functions are important given the prevalence and societal impact of brain diseases that involve the dysfunction of NVU. Furthermore, brain diseases caused by stroke, Alzheimer's disease, traumatic brain injury, and neurodegenerative disorders contribute heavily to morbidity and mortality worldwide. They pose a substantial challenge to the healthcare systems and society.

Pericytes play a key role in the NVU and are a promising target in understanding and treating neurological conditions. This can help to identify the functions and dysfunction of pericytes and facilitate the development of new therapeutic strategies for brain diseases. Consistent with the extensive distribution of microvessels in the entire body, pericytes are present on the vascular walls of almost all tissues and organs. Notably, the highest density of pericytes is in the brain tissues and the retina. The ratio of pericytes to vascular endothelial cells is approximately 1:1 in brain tissue microvessels [1, 2], significantly higher than in lung tissue (1:7-1:9) [3], liver (1:10) [4], kidney (1:2.5) [5], and heart tissue (1:2) [6]. Generally, the pericytes in the tissues contribute greatly to the establishment of BBB, which protects the brain cells from harmful blood-derived factors [7, 8]. Therefore, pericytes play a vital role in brain microvessels-related diseases.

## Overview of the Role of Pericytes in Various Brain Diseases

It is important to establish a detailed context of pericyte function in the healthy brain to provide a more comprehensive understanding for the reader. Therefore, a section dedicated to the role of pericytes in the NVU and BBB will be included. These sections contain details of their physiological functions: integrity to the BBB, regulation of cerebral blood flow, and their contribution to the stability of vessels. Additionally, the review will include a section on the role of pericytes in brain development, as it is important to understand how pericytes acquire these functions. This section will first address the role of pericytes in forming the cerebral vasculature alongside other cell types and the signaling pathways controlling the differentiation and maturation of pericytes. This review provides a solid foundation to understand the pericyte function in NVU/BBB and brain development before moving to the complexities of pericyte involvement in pathological conditions by dedicating specific sections to pericyte function in NVU/BBB and brain development. Organizing the review based on the structure described will further improve the informativeness and logical flow of the information and increase the understanding of the therapeutic potential of pericytes in treating brain diseases.

### Pericytes and Stroke

Stroke, also known as cerebrovascular accident or CVA is one of the leading causes of death and disability worldwide. With the aging population, the incidence of stroke is expected to increase progressively. Studies have found that pericytes are related to post-stroke disruptions in the BBB, central nervous system inflammation, and angiogenesis. In animal models of stroke, the number and coverage of pericytes decrease rapidly [9-11]. Enhancing pericyte presence in stroke-affected animals can aid in the restoration of compromised BBB function [12, 13].

#### Pericytes and the BBB Following Stroke

The BBB refers to the barrier formed by the walls of brain capillaries and neuroglial cells between the plasma and brain cells and the barrier between the plasma and cerebrospinal fluid formed by the choroid plexus. These barriers prevent certain substances (mostly harmful) from entering brain tissue from the bloodstream. Dysfunction of the BBB is both a result and a cause for further deterioration in stroke. The BBB primarily comprises endothelial cells, the basement membrane, pericytes, and astrocytes. Pericytes play a dual role in maintaining the BBB due to their contractile and inductive capabilities. During ischemic stroke, vessel constriction leads to the loss of pericytes, causing restricted blood flow in brain regions and the failure of BBB integrity [9-11]. On the one hand, after an ischemic stroke, pericytes regulate the integrity of the blood-brain barrier by adjusting cerebral vascular permeability and expressing molecules such as vascular endothelial growth factor and metalloproteinases [14, 15]. On the other hand, during ischemic stroke, the tight junctions between different types of cells forming the BBB are disrupted, and the expression of claudins that connect endothelial cells, pericytes, and astrocytes decreases, leading to increased BBB permeability. This allows a series of harmful substances to enter brain tissue, causing neuroinflammation and oxidative stress [16]. Moreover, in hemorrhagic stroke, the reduction of pericyte coverage on microvessels damages the integrity of brain microvessels, which can lead to the occurrence of hemorrhagic stroke [17, 18]; during the development of hemorrhagic stroke, pericytes are recruited to wrap around endothelial cells to reduce vascular permeability. However, when this recruitment effect of pericytes disappears, it leads to increased vascular permeability and severe disruption of the BBB, exacerbating the disease progression [17, 18]. Thus, it is evident that pericytes, a major component of the BBB, influence the development of stroke diseases by affecting the integrity of the BBB.

#### Pericytes and Angiogenesis Post-Stroke

The fundamental principle of stroke treatment is reestablishing collateral pathways post-infarction, which primarily depends on forming new blood vessels. Angiogenesis after cerebral ischemia mostly occurs through sprouting, encompassing initiation, progression, and formation, as well as vessel remodeling and maturation stages. Due to pericytes sharing regenerative properties similar to mesenchymal stem cells, they significantly promote vascular regeneration following a stroke. In animal models, the injection of pericytes isolated in vitro has been observed to enhance angiogenesis [19]. Previously, pericytes were believed to be mainly involved in the later stages of vessel formation [20, 21]. Still, recent studies have shown that pericyte growth precedes endothelial cell expansion during the early stages of angiogenesis[22-24], although the reasons are still unclear. Moreover, previous research indicated that in the cerebral vascular system, when pericytes are absent, endothelial cells can form vessels but fail to proliferate further [7]; rapidly proliferating pericytes are thought to provide sufficient stimulatory signals for the proliferation of endothelial cells[22]. In summary, pericytes are essential for angiogenesis.

Molecular mechanism studies have demonstrated that post-stroke, pericytes migrate towards the damaged infarct area in response to chemotactic factors such as platelet-derived growth factor β (PDGFβ) and transforming growth factor β (TGF-β). Simultaneously, endothelial cells also migrate to the vicinity of the infarct, together with pericytes, forming the basic structure of vessels through proliferation [25, 26]. Renner O et al. [25] found in a mouse model of ischemic stroke that PDGFβ and its receptor expression continuously increased in and around the infarct area; immunohistochemistry and confocal microscopy analysis revealed that PDGFβ was mainly secreted by endothelial cells of new vessels, while its receptors were primarily expressed by pericytes of new ships, facilitating pericyte migration towards endothelial cells. Based on this mechanism, Bernard M et al. [27] designed an approach in vivo to enhance the bioavailability of PDGF-D (a specific ligand for PDGFRβ), improving the association between pericytes and cerebral vascular endothelial cells, reducing pericyte loss and fibrotic changes, promoting stable neovascularization in injured tissues, and improving cerebral perfusion. Therefore, pericytes can serve as one of the therapeutic targets for promoting angiogenesis post-stroke; for instance, Cui Q et al. [28] discovered that leptin improves neurological function recovery post-intracerebral hemorrhage by promoting angiogenesis, with the mechanism being that leptin regulates the leptin receptor and transcription activator three signaling pathway in pericytes post-hemorrhage to promote angiogenesis. Additionally, Dasen B et al. [29] found that T-cadherin protein regulates pericyte proliferation, migration, invasion, and interaction with endothelial cells during angiogenesis in vitro and in vivo and developed a porous three-dimensional channel slide containing T-cadherin protein for in vitro angiogenesis analysis. In conclusion, numerous previous studies have shown that the interaction between pericytes and endothelial cells during stroke development promotes angiogenesis to help patients recover from impaired neurological functions.

#### Pericytes and Inflammation Post-Stroke

Following a stroke, the levels of reactive oxygen species in the infarcted tissue (ischemic stroke) or hemorrhagic area (hemorrhagic stroke) rapidly increase, overwhelming the body's natural antioxidant defense mechanisms. This leads to pathological damage, including inflammatory responses. During the post-stroke inflammatory cascade, pericytes play a dual role in modulating inflammation progression through two main mechanisms: firstly, by regulating their own pro-inflammatory/anti-inflammatory protein expression to influence inflammation development; secondly, by recruiting and regulating neutrophil extravasation to affect inflammation progress. On the one hand, pericytes can quickly recognize the inflammatory state within blood vessels through expressed PDGFβ receptors and inhibit the expression of inflammatory factors such as IL-1β and ICAM-1 by secreting anti-inflammatory factors like CEBPδ [30]. Moreover, after being stimulated by inflammatory signals post-stroke, pericytes synthesize and secrete pro-inflammatory factors such as CCL-2, CXCL1, CXCL8, and macrophage migration inhibitory factors [31]. On the other hand, since pericytes possess some characteristics of mesenchymal stem cells, their functions vary depending on their location; for example, pericytes in capillaries and post-capillary venules do not express or only minimally express α-smooth muscle actin (α-SMA), thus lacking the ability to constrict blood vessels but have been shown to regulate the extravasation of leukocytes from blood vessels [32, 33]. Joulia R et al. [33] found that neutrophils move from the vessel lumen towards the perivascular mast cells along pericyte processes and identified that IL-17A derived from perivascular mast cells regulates neutrophil extravasation by adjusting pericyte ICAM-1 and CXCL-1. Furthermore, inflammatory cytokines secreted by pericytes, such as ICAM-1, CXCL-1, CCL-2, and CLXL8, have a strong chemotactic effect on neutrophils [34], and studies have confirmed that during vascular inflammation, neutrophils tend to extravasate from gaps between pericytes with high expression of ICAM-1 and CXCL-1 [35, 36]. Therefore, it is evident that pericytes primarily regulate inflammatory responses after a stroke by controlling their inflammatory cytokine expression and neutrophil extravasation. However, it is important to note that current research on pericyte regulation of leukocyte extravasation mainly focuses on neutrophils, with less study on interactions with other types of leukocytes, which warrants further exploration by researchers.

### Pericytes and Traumatic Brain Injury

Traumatic brain injury (TBI) is caused by a blow or jolt to the head or body and has become one of the leading causes of death and disability among adolescents in developed countries [37, 38]. Among common neurological disorders, TBI has the highest incidence rate, posing a significant public health burden. TBI is not only an acute injury but is increasingly considered a chronic disease with long-term effects on patients, including an increased risk of late-onset neurodegenerative diseases. TBI damage includes direct brain injury and secondary brain injury involving neuroinflammation, oxidative stress, and the production of matrix metalloproteinases, all leading to BBB dysfunction [39-41], similar to brain damage caused by stroke. Zheng S et al. [42] found that TNF-α synthesized and secreted by microglia hours after TBI promotes neuroinflammation and oxidative stress through the activation of downstream NF-kB/iNOS signaling pathways, leading to pericyte-mediated brain microcirculation disorder.

Pericyte loss is another cause of secondary damage in TBI. Ojo J et al. observed a decrease in pericyte marker expression in human TBI brain specimens and in a mouse model of repetitive mild TBI within 12 months post-injury [43], and many studies have observed rapid pericyte loss in the acute phase of TBI in mouse models [44, 45], finding that pericyte loss may be related to apoptosis caused by TBI [46]. Additionally, similar to stroke, pericytes can cause brain damage in TBI through interactions with endothelial cells, mechanisms related to causing BBB dysfunction, specifically that TBI-induced pericyte loss leads to decreased secretion of PDGFβ receptors, further resulting in the loss of interaction between pericytes and endothelial cells [45]. Interestingly, Whitehead B et al. [47] found that mild TBI injury leads to pericyte detachment from the vasculature. However, they also discovered that the detachment of these pericytes following TBI did not worsen infarct volume in mice after a subsequent stroke. This implies that, in this particular setting, mild TBI does not increase susceptibility to or severity of stroke after delinking of pericytes. In summary, developing treatments that could prevent or reverse the degeneration or loss of pericytes is promising for treating TBI and successive secondary damage.

**Figure 1.**
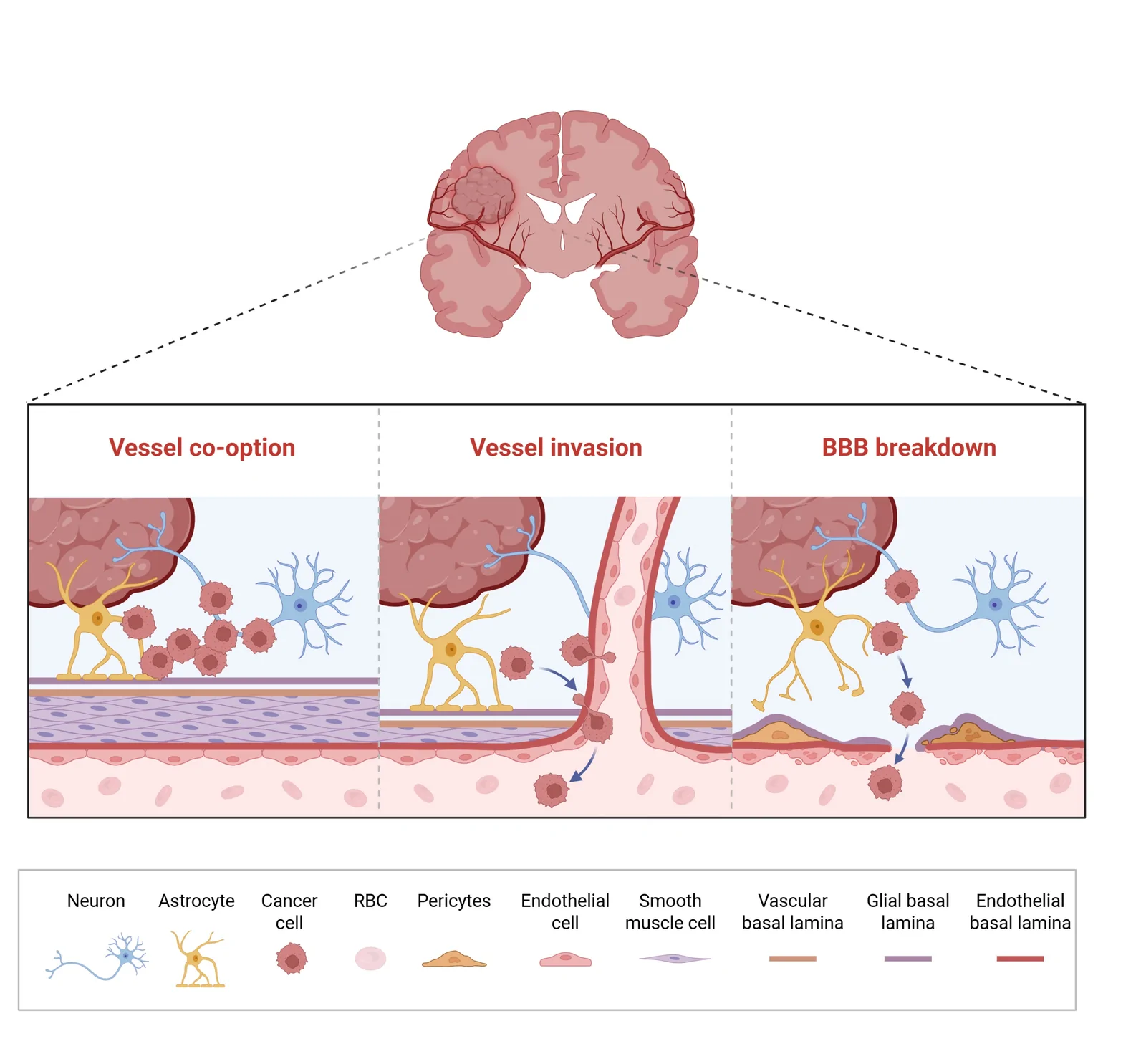
Neurovascular Units with Pericytes.

### Neurovascular Units with Pericytes

Figure 1 represents how pericytes, parts of neurovascular units (NVUs), are associated with the development and progression of brain disease. Three stages are presented in the figure: vessel co-option, vessel invasion, and BBB breakdown. The stages show how pericytes are essential for tumor cells to invade and disrupt the NVU. In the process of 'vessel co OPTION,' tumor cells move with established vasculature. Pericytes facilitate tumor cell invasion of blood vessels, which is termed 'vessel invasion'. Finally, the "BBB breakdown" shows the occurrence of a BBB compromised by pericyte loss and alterations in the NVU integrity, allowing tumor cells to invade brain tissue.

### Pericytes and Alzheimer's disease

Alzheimer's disease (AD) is a neurodegenerative disorder associated with cognitive impairment, accumulation of amyloid-beta (Aβ) protein, vascular dysfunction, and neuroinflammation. Cerebral vascular dysfunction, such as in the form of reduced cerebral blood flow and disruption of BBB, has been identified as an early critical factor in AD onset and a reliable predictor of cognitive decline [48, 49]. Notably, degeneration/loss of pericytes has been observed in AD patients and animal models. Given the crucial role of pericytes in maintaining vascular function and ensuring BBB integrity, they are considered to play a significant role in the development and progression of AD [50, 51]. Researchers have observed varying degrees of pericyte loss in AD patients across different brain regions, including white matter, precuneus, cortex, and hippocampus [52-54]. Apoptosis of pericytes is believed to be one of the causes of pericyte loss in AD animal and cellular models. Li P and Shi H [54, 55] observed pericyte apoptosis in the retina and hippocampal region of AD patients, and Aβ stimulation can induce apoptosis in cultured pericytes in vitro [54, 56]. Meanwhile, inhibiting pericyte apoptosis has become a potential strategy for preventing and treating AD. Li P et al. [54] found that Friend leukemia integration 1 (FLI-1), a member of the E26 transformation-specific transcription factor family involved in hematopoiesis and vascular endothelial cell development, was upregulated in brain tissues and associated with reduced pericyte numbers increased inflammatory mediators, and Aβ accumulation; knocking down FLI-1 by injecting small interfering RNA into the hippocampus not only delayed pericyte loss in AD mice but also reduced inflammation and Aβ accumulation, improving cognitive deficits and BBB dysfunction. Additionally, Wu Q et al. [56] first discovered decreased expression of miR-181a in the brain tissues of APP/PS1 mice, which coincided with increased Aβ levels; then, by injecting lentivirus to overexpress miR-181a into the hippocampal region of APP/PS1 mice, they found that overexpression of miR-181a not only reduced pericyte apoptosis and BBB disruption in the brain but also improved cognitive deficits and amyloid plaque production in APP/PS1 mice. However, it is important to note whether pericyte loss is a cause or a consequence of AD onset has not been fully confirmed, and interventions targeting pericyte loss have only been proven beneficial in slowing AD progression in animal models.

Another mechanism by which pericytes are involved in AD progression relates to their contractile function. Early in AD onset, reduced cerebral blood flow was discovered, and since most vascular resistance in the brain occurs at the capillary level, this suggests that dysfunction of pericytes, which perform contractile functions on the capillary walls, is related to AD onset [57]. Further mechanistic studies found that Aβ-induced neurotoxicity releases endothelin-1, which activates endothelin receptors in pericytes, causing capillary constriction [57]. Moreover, a form of AD independent of Aβ pathology has also been linked to pericytes; cognitive decline and BBB disruption in AD patients were associated with accelerated pericyte degeneration in carriers of the AD susceptibility allele APOE4 [58]; and high baseline levels of PDGFβ receptor were observed in the cerebrospinal fluid of APOE4 carriers, indicating severe pericyte loss in the BBB of these AD patients [58]. Additionally, single-cell sequencing technology and phosphoproteomics analysis of the cortex of APOE4-carrying mice revealed that signaling mechanisms in endothelial and pericyte cells were dysregulated, reflecting molecular characteristics of BBB failure before prominent functional and behavioral changes [59]. However, it should be noted that mice do not fully reproduce the features of AD, so further research is needed to confirm the AD features caused by pericyte-mediated BBB failure in APOE4 mice. In summary, pericyte-mediated vascular dysfunction and BBB disruption are pathological bases of AD onset, and inhibiting pericyte apoptosis to prevent pericyte loss is one of the potential strategies for preventing and treating AD.

#### Pericytes and Brain Tumors

Clinical research over the past few years has shown that pericyte dysfunction is associated with cancer progression [60]. It has been established that tumor vasculature is poorly covered by pericytes, and a reduction in pericyte recruitment and maturation leads to enhanced vascular and tumor growth [61, 62], with pericyte depletion facilitating tumor cell metastasis [63]. This summarizes the relationship between pericytes and cancer development, including brain tumors. In gliomas, pericytes have been found to play a pro-cancer role [64], with upregulated expression of pericyte-related markers being one of the signs of progression from low-grade gliomas [65], and pericyte proliferation being one of the malignant features of glioblastoma multiforme (GBM) [66]. Tumor progression and invasion through the perivascular space are some of the most important characteristics of GBM, as they cause vascular changes in a process known as vessel co-option, identified by vascular abnormalities at the infiltrative tumor margin. Previous studies have confirmed [67, 68] that the primary target cells of GBM cells during the process of vessel selection are pericytes and the contact between GBM cells and pericytes. Further, the subsequent activation of tumor-promoting cellular and molecular processes within pericytes is crucial for GBM cell growth and disease progression. Furthermore, pericytes have also been shown to promote glioma cell proliferation, invasion, and migration through their synthesis and secretion of osteopontin [69, 70]; Zhang XN et al. [71] discovered that pericytes enhance DNA damage repair in GBM cells in the perivascular microenvironment, thereby inducing temozolomide chemotherapy resistance. The mechanism involves pericyte-secreted CC motif chemokine ligand 5 (CCL5) activating CC motif chemokine receptor 5 (CCR5) on GBM cells, which, after temozolomide treatment, enables DNA-dependent protein kinase catalytic subunit (DNA-PKcs)-mediated DNA damage repair. Other mechanisms by which pericytes promote glioma progression include promoting the formation of abnormal vessels in glioma rat models [72], being activated by glioma cells to differentiate into immunosuppressive pericytes that promote tumor cell growth [73], and inhibiting the malignant proliferation of pericytes can improve the therapeutic efficacy of gliomas [74, 75].

**Figure 2.**
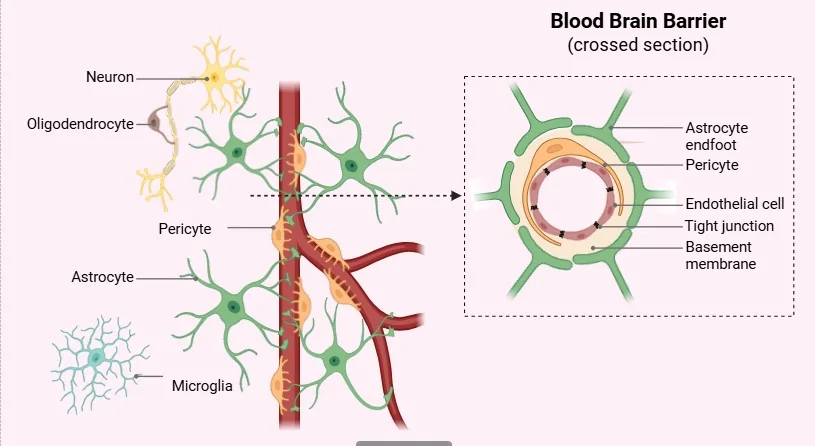
Pericyte Positioning within the Healthy Neurovascular Unit.

## Pericyte Positioning within the Healthy Neurovascular Unit

Figure 2 provides important context for understanding the role of these in the development and progression of brain diseases. It shows an arteriole branching into capillaries with the intermittent pericytes along the vessel wall. The magnified cross section of the NVU shows endothelial cells lining the lumen and basal lamina in which pericytes reside. The blood brain barrier is shown by astrocytic endfeet around the unit. This healthy configuration is something that needs to be understood. The manuscript discusses that pericyte loss, dysfunction, or altered positioning disrupts NVU integrity and BBB function, and is related to various neuropathologies. This healthy state is set up in this figure to understand how deviations from this healthy state lead to disease.

### Pericytes and Brain Tissue Infection

Brain damage caused by HIV, SARS-CoV-2, and other microbial infections is closely related to pericytes. Neurocognitive impairment is one of the main complications of HIV infection, primarily due to the rapid entry of HIV into the brain after infecting the human body, leading to neuroinflammation and disruption of BBB [76, 77]. In vitro, increased expression of inflammatory mediators and disruption of the endothelial barrier properties of pericytes were observed after HIV infection [78]. In vivo, HIV infection can be detected in brain pericytes in both humans and humanized mice [79], and a reduction in pericyte coverage has been observed in brain tissue following HIV infection [80, 81]. However, research on the role of pericytes in brain damage caused by HIV infection is still in its preliminary stages. It has not been extensively studied, with the underlying molecular mechanisms yet to be fully revealed. Similarly, SARS-CoV-2 infection in humans also affects various organ functions, including the brain [82]. Studies have found that since brain pericytes express ACE-2, they are susceptible to SARS-CoV-2 infection, leading to inflammation and vascular dysfunction [83, 84]. Additionally, Bocci M et al. [83] found decreased levels of the pericyte marker PDGFβ receptor in the cerebrospinal fluid of COVID-19 patients. Research has shown that the spike protein of SARS-CoV-2 can interact with brain pericytes to regulate vascular and immune functions [84]. In contrast, the envelope protein of SARS-CoV-2 can induce brain pericyte death in vitro [85]. However, like HIV infection, research on the role of pericytes in brain damage caused by SARS-CoV-2 infection is also in its early stages, and further in-depth study of their function is needed.

### The Intertwined Destinies of Pericyte Dysfunction, BBB Disruption, and Brain Disease

Neurological pathology revolves around the central role of pericyte dysfunction, BBB disruption and the development of and progression of several brain diseases. The significance of NVU for BBB function relies on pericytes [86]. Among these are their strategic location and diversity of function, including regulation of endothelial cell tight junctions, endothelial support of microvessels, and secretion of critical signaling molecules, all essential for the BBB function to protect the delicate neural tissue [87]. The integrity of the BBB is compromised either by malfunction or loss of pericytes, leading to or amplifying a pathologic cascade [88]. Pericyte failure in acute conditions like stroke and traumatic brain injury is extremely rapid and is associated with increased BBB permeability, vasogenic edema, increased neuroinflammation and neuronal damage.

There is increasing evidence that pericyte degeneration and functional impairment play a role in the early stages of BBB breakdown in chronic neurodegenerative diseases such as Alzheimer's. Finally, cerebral blood flow, as well as the accumulation of neurotoxic metabolites, such as amyloid-beta, are impaired by this chronic BBB leakage, contributing to cognitive decline. Similarly, pericyte abnormalities are also important for the formation of a compromised and hyperpermeable tumor vasculature in brain tumors, which promotes tumor growth, invasion, and resistance to chemotherapeutic agents. The challenge is to address this complex game of pericytes, BBB, and brain diseases. Areas of investigation consist of deciphering the specific molecular mechanisms by which pericyte dysfunction leads to BBB breakdown in the context of multiple neuropathologies, identification of markup markers for early pericyte dysfunction to allow for early diagnosis and targeted treatments and designing therapy targeting to maintain or rescue pericyte function to prevent and treat BBB damage associated neurological disorders.

### Pericyte Association within the Neurovascular Unit

**Figure 3.**
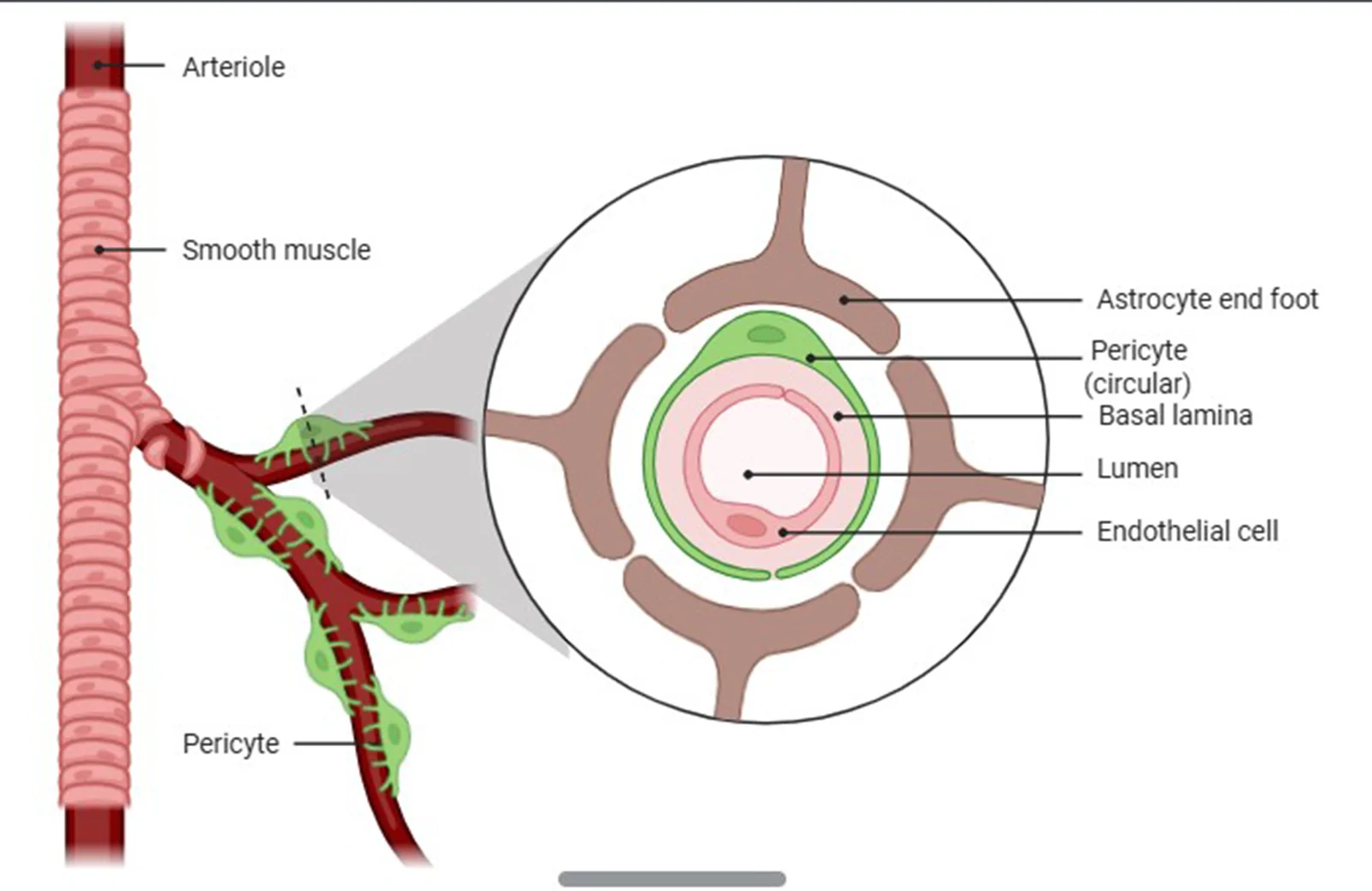
Pericyte Association within the Neurovascular Unit.

The illustrated figure 3 demonstrates the critical role of pericytes within the neurovascular unit (NVU), which is the key focus of the manuscript “Pericytes in the development and progression of brain diseases.” The NVU consists of neurons, oligodendrocytes, astrocytes, microglia and vasculature. Pericytes are also shown to be embedded within the basement membrane of a blood vessel, closely opposed to endothelial cells, forming the blood brain barrier (BBB). It is likely that the manuscript is about how pericyte dysfunction affects BBB integrity and its role in brain diseases. The image shows that pericytes and astrocyte endfeet directly contact endothelial cells and are responsible for regulating blood flow, nutrient delivery and waste removal. The manuscript probably explains that damage or loss of pericytes disrupts the BBB, causing inflammation, neuronal damage and progression of various neurological disorders. Thus, the manuscript may stress the importance of pericytes as potential therapeutic targets for brain diseases by restoring BBB function and protecting the NVU.

## Pericytes and hypertension

Hypertension is a risk factor for cardiovascular and cerebrovascular diseases [89], and long-term high blood pressure can damage the BBB, eventually resulting in diseases affecting the central nervous system (CNS) [90]. Pericytes can promote BBB function by secreting various soluble factors such as Ang-1 [91]. In addition, pericyte apoptosis increases BBB permeability, which in turn diminishes the stability of the brain microenvironment, possibly resulting in the development and progression of brain diseases [92, 93]. Our team's research revealed that Salvianolic Acid alleviated the BBB permeability of SHR rats, and the mechanism was to reduce the apoptosis of pericytes [94]. Some lncRNAs are considered biomarkers and therapeutic targets in cancer, cardiovascular disease and pathophysiology [95-97]. Our study highlighted novel functions of lncRNAs in a gene regulation network focused on microcirculation, a dynamic that is vital to hypertension [98]. Extracellular vesicles (EVs) are released from most eukaryotic cells and contain lipids, proteins, mRNAs and miRNAs [99]. Endothelial cells can easily take up EVs derived from pericytes, which are another important component of the neurovascular unit [100]. By using miRNA profiling analysis, our research explored EVs' differential expression patterns in brain microvascular pericytes from SHR and WKY rats. Several EV miRNAs in brain microvascular pericytes were identified as potential biomarkers or therapeutic targets for hypertension [101]. Our study also revealed the m^6^A landscape, identifying the epitranscriptomic mechanisms during mammalian hypertension development. It suggested that the pathogenesis of hypertension potentially involves the changes in m^6^A methylation level occurring in microvascular pericytes [102].

## Emerging Pericyte Identification Strategies

Traditional markers for pericytes, such as α-smooth muscle actin (α-SMA), platelet-derived growth factor receptor β (PDGFRβ), and desmin, are used frequently. Still, lack of exclusivity to pericytes, presence of heterogeneity according to tissue type, tissue activation or disease state prevent usage of such markers as a sole identifier of pericytes. This heterogeneity underlines the need for further well-defined and specific strategies to identify pericytes.

Addressing this challenge will require emerging strategies to cover, which approach them in a multifaceted way. Single-cell RNA Sequencing (scRNA-seq) as a powerful tool in profile gene expression at the individual cell level, has exposed that pericyte subpopulations can be distinguished from other cells in the neurovascular unit by their unique transcriptional signatures. This approach can be used to define a complete and more precise pericyte identity by expressing a set of genes. Additionally, novel fluorescent reporters and genetically encoded tools will be developed to be selective to pericytes based on their unique molecular profiles. Cells like pericytes can be used in studying their dynamic behavior and interactions, and these tools would allow for their specific labeling and tracking in vivo. A promising alternative entails using advanced imaging techniques and machine learning algorithms to distinguish pericytes from their morphology and spatial relations with the microvasculature. Regarding that, such an approach can supplement molecular marker identification and help to characterize pericytes better. Eventually these newly emerging strategies will have to be brought into unification in order to have a greater understanding of pericyte biology and their roles in maintaining healthy brain processing and various disease states.

### Pericyte Influence on Endothelial Cell Tight Junction Expression and BBB Function.

**Figure 4.**
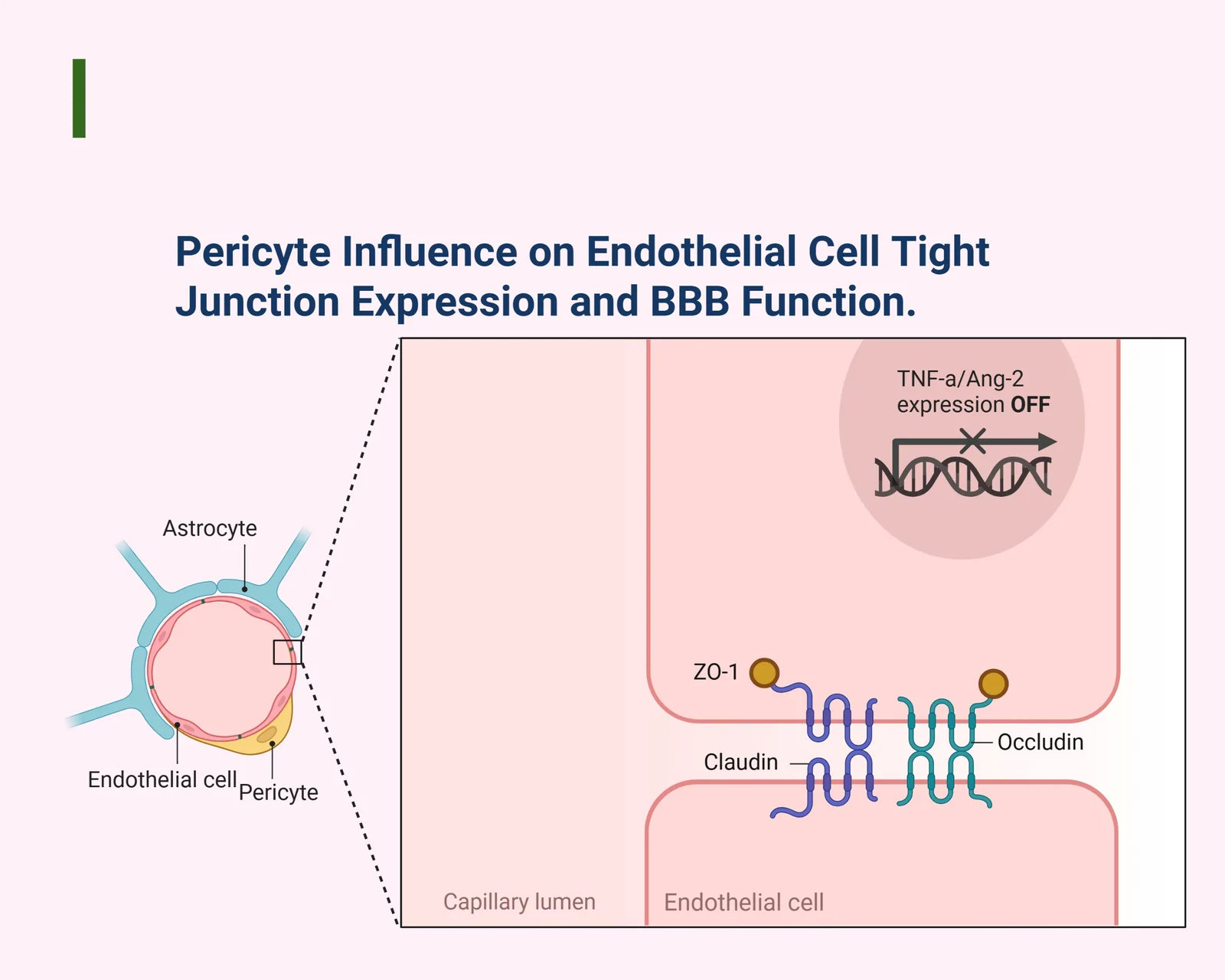
Pericyte influence on endothelial cell tight junction expression and BBB function.

Figure 4 visually supports the central theme of the manuscript: "Development and progression of brain diseases requires pericytes." This shows the essential BBB-maintaining role of pericytes and the way pericytes are dysregulated in many neurological conditions. Here we begin magnifying the view on the tight junctions between endothelial cells caused by proteins like claudin, occludin, and ZO-1. The selective permeability of the BBB depends on these junctions. The image indicates "TNF-α/Ang-2 expression OFF" indicating that pericytes suppress the expression of these inflammatory mediators. Being well known to disrupt tight junctions, thereby increasing BBB permeability and promoting neuroinflammation and disease progression, TNFα or Ang2. The image thus suggests that pericytes prevent TNF-α/Ang-2 from inhibiting the expression and proper assembly of tight junction proteins. It is important for a healthy BBB and protecting the brain from harmful substances. It is likely that the manuscript discusses how pericyte dysfunction, with increased TNF-α/Ang-2 expression and compromised tight junctions, is involved in the pathogenesis of brain diseases. Restoration of BBB integrity that would mitigate disease progression could be achieved by targeting pericyte function.

## Therapeutic Opportunities Targeting Pericytes

Therapeutic strategies can be divided into attempts to protect and stabilize pericytes and interventions aimed at regulating their function. Therapeutic strategies aimed at preventing pericyte loss or degeneration in conditions where such pericyte degeneration or loss occurs, such as in stroke and Alzheimer's disease, are of greatest importance. Incorporating this would require the delivery of growth factors to PDGFβ or the administration of pericyte inhibitors. Additionally, strategies to restore pericyte–endothelial cell interactions and restore BBB integrity may be effective in improving cerebral blood flow.

On the contrary, treating pericytes may be beneficial in conditions like brain tumors, where pericytes are necessary for tumor progression angiogenesis, as therapy may focus on inhibiting pericyte proliferation, migration, or secretion of pro-tumorigenic factors. First, although the majority of anti-angiogenic therapies target endothelial cells, they also indirectly affect the pericytes; second, cellular strategies that mediate pericyte tumor cell interactions may typically provide new therapeutic opportunities. Yet, as is often the case, while it is important to acknowledge that the pericyte-targeted therapies are still in their infancy, more research is critical to optimize treatment strategies and make them safe and effective. Nevertheless, the revised knowledge of the biology of pericytes provides a compelling rationale to explore these therapeutic opportunities to treat many types of neurological disorders.

## Future Directions and Emerging Perspectives in Pericyte Research

Though much progress has been made in uncovering the functions of pericytes in several forms of brain disease, the field is in its infancy, and there are many avenues for future investigation. Refinement of methodologies and techniques to more dynamically and precisely proscribe the pericyte behavior in vivo constitutes one area that needs to be developed. Current techniques usually stop just at static snapshots rather than real-time snapshots that can provide the interactions of different pericytes within NVUs over time and in response to different stimuli. However, blocking these limitations will be difficult without advanced imaging modalities such as two-photon microscopy with genetically coded sensors and microfluidics that mimic the neurovascular niche that allows live, real-time analysis of pericyte activity, migration, and signaling. Additionally, such integration of multi-omics advances an exciting frontier for further understanding pericyte biology. The fact that pericyte functions are complicated and often occur in a heterogenous manner makes single-cell transcriptomics, proteomics and metabolomics an ideal tool to give insights into pericyte heterogeneity, signaling pathways and molecular signatures, either healthy or diseased. If such comprehensive analyses identified novel therapeutic targets and biomarkers of early disease identification and intervention, it would be repeatedly useful in any future patient of this series. Since pericyte research in the context of brain diseases is in its infancy, several key research questions should be prioritized. These include precisely defining the mechanisms through which pericytes contribute to the pathogenesis of specific brain disorders, identifying reliable and specific markers for pericytes to facilitate their unambiguous identification and study, elucidating the complex interplay between pericytes and other cellular components of the neurovascular unit, including endothelial cells, astrocytes, and immune cells, and developing targeted therapeutic strategies that can selectively modulate pericyte function to restore neurovascular health and improve patient outcomes. The resolution of these challenges is expected to accelerate the translation of pericyte research into clinically relevant assessments and enable the development of new treatments for devastating brain diseases across the spectrum.

## Conclusion

Brain tissue is one of the two tissues with the highest density of pericyte distribution. Changes and losses in its function play a significant role in BBB disruption caused by stroke, traumatic brain injury, Alzheimer's disease, brain tumors and hypertension. Strategies for treating these diseases using pericytes are gradually being developed and further validated. However, due to the lack of specific markers, the identification of pericytes is a major obstacle to their in-depth study. The mechanism by which pericytes regulate inflammation in many diseases has not been fully revealed, and their interaction mechanisms with leukocyte subtypes other than neutrophils are also blank. In addition, the relationship between brain damage caused by viral or other microbial infections and pericytes is still in the preliminary stage, with many mechanisms remaining unclear. In summary, pericytes play an important role in many brain diseases. However, due to the lack of specific markers, most research on brain diseases involving pericytes is still in its early stages.
